# Complete genome sequences of two *Escherichia coli* clinical isolates from Egypt carrying *mcr-1* on IncP and IncX4 plasmids

**DOI:** 10.3389/fmicb.2022.989045

**Published:** 2022-09-09

**Authors:** Ahmed M. Soliman, Hazem Ramadan, Liansheng Yu, Junzo Hisatsune, Motoyuki Sugai, Shimaa S. Elnahriry, Hirofumi Nariya, Ramadan A. El-Domany, Toshi Shimamoto, Charlene R. Jackson, Tadashi Shimamoto

**Affiliations:** ^1^Department of Microbiology and Immunology, Faculty of Pharmacy, Kafrelsheikh University, Kafr El Sheikh, Egypt; ^2^Hygiene and Zoonoses Department, Faculty of Veterinary Medicine, Mansoura University, Mansoura, Egypt; ^3^Bacterial Epidemiology and Antimicrobial Resistance Research Unit, U.S. National Poultry Research Center, U.S. Department of Agriculture, Agricultural Research Service (USDA-ARS), Athens, GA, United States; ^4^Antimicrobial Resistance Research Center, National Institute of Infectious Diseases, Higashimurayama, Japan; ^5^Department of Antimicrobial Resistance, Graduate School of Biomedical and Health Sciences, Hiroshima University, Hiroshima, Japan; ^6^Department of Bacteriology, Mycology and Immunology, Faculty of Veterinary Medicine, University of Sadat City, Sadat City, Egypt; ^7^Laboratory of Food Microbiology, Graduate School of Human Life Sciences, Jumonji University, Niiza, Japan; ^8^Laboratory of Food Microbiology and Hygiene, Graduate School of Integrated Sciences for Life, Hiroshima University, Higashihiroshima, Japan

**Keywords:** *E. coli*, Egypt, IncP plasmid, IncX4 plasmid, ST1011, ST744, *mcr-1*

## Abstract

Colistin is a last-resort antibiotic used in the treatment of multidrug resistant Gram-negative bacteria. However, the activity and efficacy of colistin has been compromised by the worldwide spread of the mobile colistin resistance genes (*mcr-1* to *mcr-10*). In this study, two clinical *Escherichia coli* strains, named *Ec*CAI51, and *Ec*CAI73, harbored *mcr-1*, showed multidrug-resistant phenotypes (with colistin MIC = 4 μg/ml), and belonged to phylogroup D: multilocus sequence type 1011 (ST1011) and phylogroup A: ST744, respectively. Findings revealed the existence of *mcr-1* gene on two conjugable plasmids, pAMS-51-MCR1 (∼122 kb IncP) and pAMS-73-MCR1 (∼33 kb IncX4), in *Ec*CAI51, and *Ec*CAI73, respectively. The *mcr-1*-*pap2* element was detected in the two plasmids. Additionally, the composite transposon (IS*Apl1*-IS*5D*-*pap2*-*mcr-1*-IS*Apl1*) was identified only in pAMS-51-MCR1 suggesting the potential for horizontal gene transfer. The two strains carried from 16 to 18 different multiple acquired antimicrobial resistance genes (ARGs). Additionally, two different multireplicon virulence plasmids (∼117 kb pAMS-51-Vr and ∼226 kb pAMS-73-Vr) carrying the *sit* operon, the Salmochelin siderophore *iroBCDE* operon and other several virulence genes were identified from the two strains. Hierarchical clustering of core genome MLST (HierCC) revealed clustering of *Ec*CAI73, and *Ec*CAI51 with global *E. coli* lineages at HC levels of 50 (HC50) to 100 (HC100) core genome allelic differences. To the best of our knowledge, this study presented the first complete genomic sequences of *mcr-1*-carrying IncP and IncX4 plasmids from human clinical *E. coli* isolates in Egypt. In addition, the study illustrated the *mcr-1* broad dissemination in diverse plasmids and dissimilar *E. coli* clones.

## Introduction

Colistin is one of the last-resort antibiotics used in the treatment of infections caused by multidrug or carbapenem resistant Gram-negative bacteria. In 2016, the first mobile colistin resistance gene (*mcr-1*) was reported from *Escherichia coli* and *Klebsiella pneumoniae* isolated from patients, food, and animals in China (Liu et al., 2016). *mcr-1* acts by modifying the lipid A part of the lipopolysaccharide in Gram-negative bacteria by adding phosphoethanolamine, reducing the binding affinity to colistin (Liu et al., 2016). Furthermore, *mcr-1*-carrying *E. coli* strains have been reported in Egypt from patients (Elnahriry et al., 2016), cattle, and chickens (Elbediwi et al., 2019). *mcr-1* has been detected in several plasmid groups, including IncX4, IncHI2, IncI2, IncI1, IncN, IncFIB, IncP, and IncW (Lu et al., 2018; Elbediwi et al., 2019; Soliman et al., 2021). Ten *mcr* genes (*mcr-1–mcr-10*) have been characterized, all of which confer resistance to colistin by the same mechanism described above. We reported the first *mcr-9*-carrying *Enterobacter hormaechei* clinical isolate in the Middle East (Soliman et al., 2020a). Recently, Tartor et al. (2021a) reported the first emergence of an Egyptian *K. pneumoniae* isolate co-harboring *mcr*-*10* and *fosA5* genes from bovine milk in Middle East. Other variants of *mcr* including *mcr-1*, *mcr-2, mcr-3, mcr-4*, and *mcr-7* were also reported in Gram-negative bacteria (*E. coli*, *K. pneumoniae*, and *Pseudomonas aeruginosa*) isolated from bovine milk in Egypt (Tartor et al., 2021b). We additionally reported two *mcr-1*-, *tet*(X7)-, and *fosA3*-positive *E. coli* ST155 strains showing resistance to last resort antibiotics (such as colistin, and tigecycline) from poultry farm in Egypt (Soliman et al., 2021). Recently, a uropathogenic *E. coli* strain carried *mcr-1.1* on a self-transmissible IncHI2 plasmid from Alexandria, Egypt (Zakaria et al., 2021).

Little is yet known about the genomic characteristics of *mcr-1*-carrying clinical *E. coli* strains in Egypt. Therefore, we aimed, in this study, to characterize the complete genomic sequences of *mcr-1*-carrying IncP and IncX4 plasmids from two clinical *E. coli* isolates and to perform phylogenetic analysis for these two strains.

## Materials and methods

### Bacterial strains used in this study

The two *mcr-1*-positive *E. coli* isolates, named *Ec*CAI51 and *Ec*CAI73, were detected from two patients in two different hospitals located in Cairo, Egypt. The strain *Ec*CAI51 was isolated from the eye swab of a 50-years-old male patient diagnosed with a respiratory infection in April 2015, while strain *Ec*CAI73 was isolated from a blood sample of a patient in May 2015. The two strains were identified by 16S rRNA gene sequencing using primers 27F and 1492R and screened by PCR for mobile colistin-resistance genes (*mcr-1–mcr-5*) (Table 1; Luo et al., 2015; Elnahriry et al., 2016; Liu et al., 2016; Xavier et al., 2016; Borowiak et al., 2017; Carattoli et al., 2017; Yin et al., 2017), extended-spectrum β-lactamases, carbapenemase-encoding genes, plasmid-mediated quinolone-resistance genes, and 16S rRNA methylases as previously described (Jousset et al., 2019; Soliman et al., 2020b).

**TABLE 1:** Primers used in this study for PCR screening of mobile colistin resistance (*mcr*) genes.

Primer	Nucleotide sequence (5′→3′)	Annealing Tm, target size	References
27F	GAGTTTGATCMTGGCTCAG	50°C, ∼1,600 bp	Luo et al., 2015
1492R	ACGGGCGGTGTGTRC		
CLR5-F	CGGTCAGTCCGTTTGTTC	53°C, 308 bp	Liu et al., 2016
CLR5-R	CTTGGTCGGTCTGTAGGG		
MCR-1-F2	CTCATGATGCAGCATACTTC	53°C, 1,626 bp	Elnahriry et al., 2016
MCR-1-R2	CGAATGGAGTGTGCGGTG		
MCR2-IF	TGTTGCTTGTGCCGATTGGA	65°C, 566 bp	Xavier et al., 2016
MCR2-IR	AGATGGTATTGTTGGTTGCTG		
mcr-2 full Fw	ATGACATCACATCACTCTTGG	52°C, 1,617 bp	Liassine et al., 2016
mcr-2 full Rv	TTACTGGATAAATGCCGCGC		
MCR3-F	TTGGCACTGTATTTTGCATTT	50°C, 542 bp	Yin et al., 2017
MCR3-R	TTAACGAAATTGGCTGGAACA		
Mcr-4 FW	ATTGGGATAGTCGCCTTTTT	45°C, 487 bp	Carattoli et al., 2017
Mcr-4 RV	TTACAGCCAGAATCATTATCA		
MCR5_fw	ATGCGGT TGTCTGCATTTATC	50°C, 1,644 bp	Borowiak et al., 2017
MCR5_rev	TCATTGTGGTTGTCCTTTTCTG		

### Antimicrobial susceptibility testing

The broth microdilution assay (BMD) was performed to determine the minimum inhibitory concentration (MIC) of various antimicrobials (Table 2) according to the standards and interpretive criteria described by the Clinical and Laboratory Standards Institute (Clinical and Laboratory Standards Institute [CLSI] (2020) document M100-S24) and European Committee on Antimicrobial Susceptibility Testing (EU-CAST) (for colistin and tigecycline breakpoints).^1^ For all experiments, the purified powder of each antibiotic was diluted following CLSI recommendations. *E. coli* ATCC 25922 was used as a control.

**TABLE 2:** Minimum inhibitory concentrations (MICs) for *mcr-1*-carrying strains of *E. coli* and its transconjugants identified in this study.

Strain	MIC*^a^* (μ g/ml)											
	MEM	DOR	CHL	AMP	CTX	CST	PLB	GEN	KAN	TET	CIP	NAL
*Ec*CAI51	0.25 S	0.0312 S	512 R	512 R	512 R	4 R	4 R	4 S	512 R	128 R	32 R	> 512 R
*Ec*CAI51-Tc1	1 S	1 S	128 R	64 R	< 0.25 S	2 I	4 R	1 S	4 S	32 R	0.25 S	4 S
*Ec*CAI73	0.0625 S	0.25 S	512 R	512 R	< 0.25 S	4 R	4 R	64 R	512 R	128 R	16 R	> 512 R
*Ec*CAI73-TC3	1 S	0.25 S	8 S	32 R	< 0.25 S	4 R	4 R	2 S	4 S	0. 5 S	< 0.25 S	2 S
*E. coli* ATCC25922	0.0625 S	0.0625 S	8 S	64 R	< 0.25 S	0.5 S	< 0.25 S	2 S	4 S	< 0.25 S	<0.25 S	1 S

^*a*^indicated the abbreviations of antibiotics. AMP, ampicillin; DOR, doripenem; PLB, polymyxin B; KAN, kanamycin; CTX, cefotaxime; CHL, chloramphenicol; CIP, ciprofloxacin; CST, colistin; GEN, gentamicin; MEM, meropenem; NAL, nalidixic acid; TET, tetracycline; S, sensitive; I; intermediate, R, resistant.

### Filter-mating conjugation

A mating-out assay was completed at 37°C using the two *E. coli* strains and the AZ*^r^* (azide resistant) *E. coli* J53 strain as the donor and recipient, respectively (Soliman et al., 2020b,^2021^). These experiments were performed on a solid media using filters with a 1:1 donor: recipient ratio. After a 5-h incubation, filters were resuspended in 3 ml LB broth, and bacterial mixtures were overlaid onto agar plates supplemented with colistin (2 μg/ml) and sodium azide (150 μg/ml). Colony-direct PCR was performed using CLR5-F and CLR5-R primers (Table 1) to confirm the transfer of the plasmid carrying *mcr-1*.

### Plasmid analysis, PCR-based replicon typing, multi-locus sequence typing, and *Escherichia coli* phylogroup

Plasmid analysis of the wild strains and transconjugants was performed by alkaline lysis method and PCR-based replicon typing (PBRT) (Carattoli et al., 2005; Soliman et al., 2020b). Multi-locus sequence typing (MLST) was performed for *E. coli* [using Achtman seven housekeeping genes (*adk*, *fumC*, *icd*, *purA*, *gyrB*, *recA*, and *mdh*)] according to the MLST database.^2^ *E. coli* phylogroups (A, B1, B2, and D) were detected by Triplex PCR after amplification of *chuA* and *yjaA* and the DNA fragment TSPE4.C2 as previously described (Clermont et al., 2000).

### Complete genome sequencing, and analysis

The Qiagen Genomic-tip 20/G kit (Qiagen) was used to extract the total genomic DNA following the manufacturer’s recommendations. For Illumina sequencing by MiniSeq, a Nextera XT Library Prep Kit and a Nextera XT Index Kit was used to prepare the DNA library (Illumina, San Diego, CA, United States) according to the manufacturer’s instructions. For Nanopore sequencing by GridION, construction of the library was performed by the SQK-RBK004 Rapid Barcoding Kit (Oxford Nanopore Technologies, Oxford, United Kingdom). The library was loaded onto a FLO-MIN106 R9.4.1 flow cell and sequenced with the GridION device (Oxford Nanopore Technologies, Oxford, United Kingdom). A hybrid assembly of MiniSeq short reads and Nanopore long reads was achieved by Unicycler (Wick et al., 2017). The annotation was performed using DFAST.^3^ The complete genome sequences of the two *E. coli* strains were investigated at the Center for Genomic Epidemiology^4^ using ResFinder-4.1 (identity threshold for gene predictions was 90%), MLST 2.0, pMLST 2.0, VirulenceFinder-2.0 and PlasmidFinder-2. Genomic comparisons were performed using the BRIG tool^5^ and EasyFig tool.^6^ The BLAST program^7^ and ISfinder^8^ were used to analyze the plasmids.

### Phylogenetic analyses of the *mcr-1*-positive *Escherichia coli* isolates

Raw Fastq files of the sequenced two *E. coli* strains, *Ec*CAI51 and *Ec*CAI73 were imported into Enterobase^9^ for WGS-based phylogenetic analysis. Two sets of publicly available genomes of *E. coli* in Enterobase that represent different sources and belong to sequence types (ST) ST744 (*n* = 181) and ST1011 (*n* = 157) were chosen for the analysis. Our *Ec*CAI51 (ST1011) and *Ec*CAI73 (ST744) genomes were compared separately to the selected genomes from Enterobase belonging to the same ST using single nucleotide polymorphisms (SNPs) and hierarchical clustering (HierCC) of core genome (cg) MLST (Zhou et al., 2020). *E. coli* K-12 MG1655 was used as the reference strain for SNPs analysis of isolates. Metadata for the selected genomes from Enterobase are given in Supplementary Tables 1, 2.

### Nucleotide sequence accession numbers

The complete genome sequence of *Ec*CAI51 and *Ec*CAI73 were submitted to DDBJ/ENA/GenBank under BioProject ID: PRJDB11824 (SRA accession numbers DRA012212, and DRA012213, respectively).

## Results and discussion

### Characterization of *Escherichia coli* strains EcCAI51, and EcCAI73

Two polymyxin resistant *E. coli* isolates were identified from two different hospitals located at the capital city of Egypt. The two isolates showed multidrug-resistant phenotypes. Both the isolates were resistant to colistin (MIC = 4 μg/ml), polymyxin B (MIC = 4 μg/ml), ampicillin, chloramphenicol, tetracycline, kanamycin, and fluoroquinolones but were sensitive to meropenem and doripenem (Table 2). Although both isolates were susceptible to carbapenem, carbapenem-resistant *E. coli* carrying *mcr* genes were reported (Paveenkittiporn et al., 2021). In that study, the investigators identified nine colistin and carbapenem resistant MCR and NDM or OXA-48-like-producing *E. coli* strains isolated from clinical samples in Thailand during 2016–2019 (Paveenkittiporn et al., 2021). *Ec*CAI51 was resistant to cefotaxime due to production of CTX-M-14. PCR and DNA sequencing confirmed the presence of *mcr-1* in both isolates. There was no clonal relationship between the two isolates that assigned to two different ST (ST1011 or ST744), and two different phylogenetic groups (D or A). ST1011 and ST744 had the same ST of *mcr-1*-positive clinical *E. coli* previously identified from Egypt and Denmark, respectively (Hasman et al., 2015; Elnahriry et al., 2016), and differed from the STs recognized in *mcr-1*-positive clinical *E. coli* isolates from Cambodia (ST354) (Stoesser et al., 2016) and South Africa (ST10, ST1007, ST624, ST57, ST101, ST624, and ST226) (Poirel et al., 2016). The two strains carried from 16 to 18 different multiple acquired antimicrobial resistance genes (ARGs) located on the chromosome and/or different plasmids (Table 3).

**TABLE 3:** Features of chromosome and the plasmids of *E. coli* strains *Ec*CAI51, and *Ec*CAI73 isolated from clinical samples in Egypt.

Sample	Size (bp)	GC%	No. of CDSs	MLST or pMLST	Incompatibility group	Antimicrobial resistance genes	QRDR point mutations	Virulence genes
***E. coli Ec*CAI51**								
Chromosome	4,977,650	50.6	4,540	ST1011	ND	*mdf*(A), *aph(3”)-Ib*, *aph(6)-Id*, *aadA2*, *aph(3’)-Ia*, *sul1*, *sul2*, *dfrA12*, *bla*_TEM–1B_, *bla*_CTX–M–14b_	*-parC*: S80I. *-gyrA*: S83L, D87N.	*air*, *chuA*, *eilA*, *gad*, *ireA*, *papA_F20*, *papC*, *terC*
pAMS-51-MCR1	121,922	49.2	121	ND	IncP	*aadA2b*, *aadA1*, *mcr-1.1*, *sul3*, *cmlA1*	NA	ND
pAMS-51-Vr	117,096	50.9	119	F24:A^–^:B1	IncFII: IncFIB (AP001918)	*tet*(A)	NA	*etsC*, *hlyF*, *iroN*, *iss*, *ompT*, *traT*, *sitABCD*
pAMS-51-IncI1	111,134	51	117	ST12	IncI1-Iγ	*floR*	NA	ND
***E. coli Ec*CAI73**								
Chromosome	4,728,273	50.7	4,396	ST744	ND	*mdf*(A), *aph(3’)-Ia*, *aph(3”)-Ib*, *aph(6)-Id*, *aadA5*, *mph*(A), *sul2*, *sul1*, *dfrA17*, *tet*(B), *catA1*, *bla*_TEM–1B_, *qacEΔ1*	*-parC*: S80I, A56T. *-gyrA*: S83L, D87N.	*gad*, *iha*, *mchB*, *mchC*, *mchF*, *terC*
pAMS-73-Vr	226,439	49.5	244	F18:A6:B40	IncFIA: IncFIB (AP001918): IncFIC: IncFII(K)	ND	NA	*cba*, *cma*, *cvaC*, *etsC*, *hlyF*, *iroN*, *iss*, *iucC*, *iutA*, *mchF*, *ompT*, *traT*, *tsh*, *sitABCD*
pAMS-73-IncI1	110,843	50	120	ST80 (CC31)	IncI1-Iγ	*aac(3)-IId*, *aadA22*	NA	ND
pAMS-73-IncY	110,329	46.9	110	ND	IncY	ND	NA	ND
pAMS-73-MCR1	33,304	41.9	39	ND	IncX4	*mcr-1.1*	NA	ND

gad, glutamate decarboxylase; lpfA, long polar fimbriae; iroN, enterobactin siderophore receptor protein; iss, increased serum survival; QRDR, quinolone resistance-determining region; ND, not determined; cba, colicin B; cma, colicin M; cvaC, microcin C; etsC, putative type I secretion outer membrane protein; hlyF, hemolysin F; iha, adherence protein; iroN, Enterobactin siderophore receptor protein; iss, increased serum survival; iucC, aerobactin synthetase; iutA, ferric aerobactin receptor; mchB, microcin H47 part of colicin H; mchC, MchC protein; mchF, ABC transporter protein MchF; ompT, outer membrane protease (protein protease 7); sitA, ron transport protein; terC, tellurium ion resistance protein; traT, outer membrane protein complement resistance; tsh, temperature-sensitive hemagglutinin; air, enteroaggregative immunoglobulin repeat protein; chuA, outer membrane hemin receptor; eilA, Salmonella HilA homolog; ire, siderophore receptor; papA_F20, major pilin subunit F20; papC, outer membrane usher P fimbriae.

We obtained high-quality assemblies by combining the Illumina MiniSeq short reads and the Oxford Nanopore long reads adequately for completing the genomes and the plasmids contained in both isolates (Table 3). *E. coli Ec*CAI51, and *Ec*CAI73 carried three, and four plasmids, respectively (Table 3). The chromosome of the *Ec*CAI51 strain was 4,977,650 bp in size with an average G + C content of 50.6% determining 4,540 coding sequences. ResFinder identified several chromosomal ARGs as follow: *mdf*(A), *aph(3”)-Ib*, *aph(6)-Id*, *aadA2*, *aph(3’)-Ia*, *sul1*, *sul2*, *dfrA12*, *bla*_TEM–1B_, and *bla*_CTXM–14b_. In addition, *Ec*CAI73 has 4,728,273 bp chromosome with an average G + C content of 50.7% and 4,396 coding sequences. The chromosomal ARGs in *Ec*CAI73 were *mdf*(A), *aph(3’)-Ia*, *aph(3 “)-Ib*, *aph(6)-Id*, *aadA5*, *mph*(A), *sul2*, *sul1*, *dfrA17*, *tet*(B), *catA1*, *bla*_TEM–1B_, and *qacEΔ1*.

### Identification of IncP and IncX4 plasmids carrying *mcr-1* in Egyptian clinical *Escherichia coli* isolates

**FIGURE 1:**
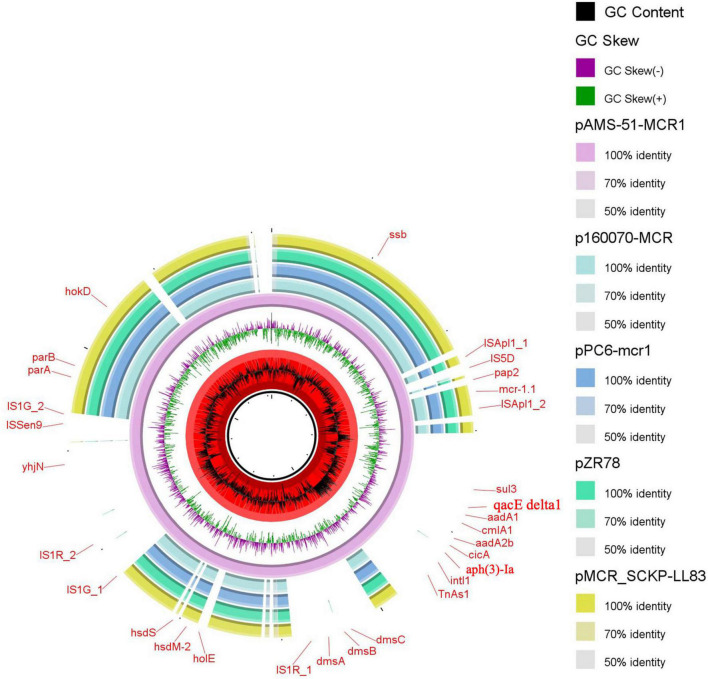
Schematic representation of IncP plasmids carrying *mcr-1.1* identified from the genome sequences of *K. pneumoniae* and *E. coli* strains analyzed in this study. Four IncP plasmid, p160070-MCR, pMCR_SCKP-LL83, pZR78, and pPC6-mcr1 carrying *mcr-1.1* (accession no. MG288678.1, MF510496.1, MF455226.1, and CP080254.1, respectively) have been detected from NCBI GenBank and was included in the figure. The whole sequence of pAMS-51-MCR1 was used as the reference. The external ring represents the annotation of pAMS-51-MCR1. The plasmids were included in the following order: pAMS-51-MCR1 (identified in this study), p160070-MCR, pPC6-mcr1, pZR78, and pMCR_SCKP-LL83.

The *mcr-1.1* gene was located on the plasmids pAMS-51-MCR1 and pAMS-73-MCR1 from isolates *Ec*CAI51 and *Ec*CAI73, respectively. pAMS-51-MCR1 was 121,922 bp IncP type (Figure 1). A BLASTn search using the whole pAMS-51-MCR1 sequence query detected that it has high identity to other *mcr-1*-carrying plasmids. for example, pAMS-51-MCR1 showed > 98.7% sequence identity to *K. pneumoniae* plasmid pSCKLB684-mcr (55% coverage; MH781719.1, IncY type), plasmid p160070-MCR isolated from food in China (56% coverage; MG288678.1, IncP type), and plasmid pMCR_SCKP-LL83 isolated from human in China (56% coverage; MF510496.1, IncP type), which were harbored *mcr-1* (Figure 1). In addition, pAMS-51-MCR1 showed > 98.7% sequence identity to *E. coli* plasmid pZR78 (56% coverage; MF455226.1, IncP type), and plasmid pPC6-mcr1 (56% coverage; CP080254.1, IncP type).

**FIGURE 2:**
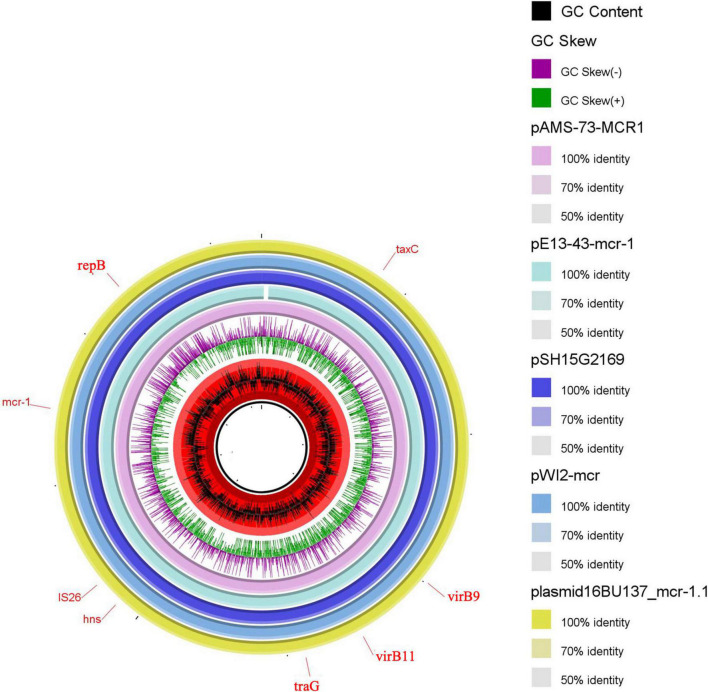
Schematic representation of IncX4 plasmids carrying *mcr-1.1* identified from the genome sequences of *E. coli*, *K. pneumoniae* or *Salmonella enterica* strains analyzed in this study. Four IncX4 plasmid, pE13-43-mcr-1, pSH15G2169, pWI2-mcr, and plasmid 16BU137_mcr-1.1 carrying *mcr-1.1* (accession no. MG747473.1, MH522417.1, LT838201.1, and MT316509.1, respectively) have been detected from NCBI GenBank and was included in the figure. The whole sequence of pAMS-73-MCR1 was used as the reference. The external ring represents the annotation of pAMS-73-MCR1. The plasmids were included in the following order: pAMS-73-MCR1 (identified in this study), pE13-43-mcr-1, pSH15G2169, pWI2-mcr, and plasmid 16BU137_mcr-1.1.

pAMS-73-MCR1 was 33,304 bp IncX4 type (Figure 2). A BLASTn search using the whole pAMS-75-MCR1 sequence query detected that it has 99.9% identity with 99% coverage to other *mcr-1*-carrying IncX4 plasmids as follow: (i) plasmid pWI2-mcr detected from clinical *E. coli* isolate WI2 in France (LT838201.1), (ii) plasmid pSH15G2169 from *Salmonella enterica* subsp. *enterica* serovar Typhimurium strain SH15G2169 isolated from diarrheal outpatients in Shanghai, China (MH522417.1) (Lu et al., 2019), iii) plasmid 16BU137_mcr-1.1 from clinical *K. pneumoniae* strain 16BU137 in China (MT316509.1), and iv) plasmid pE13-43-mcr-1 (MG747473.1) isolated from *E. coli* strain 13–43 collected from urine sample in China in 2013.

**FIGURE 3:**
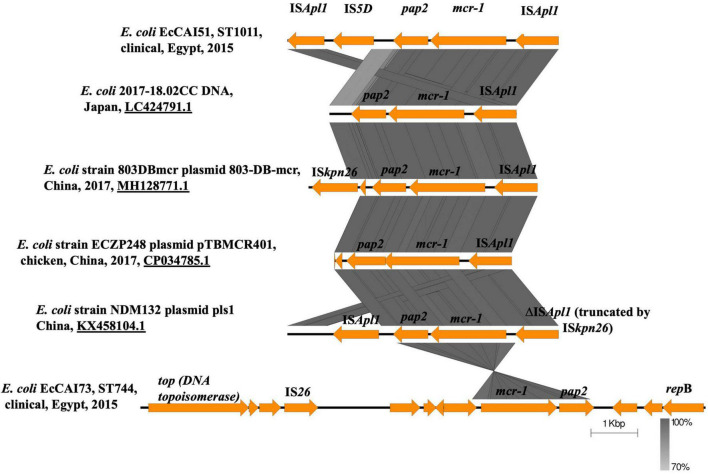
Linear comparison of the genetic environment of *mcr-1.1* detected in this study from the two strains with other *mcr-1* context from different plasmids and strains identified from NCBI GenBank.

Regarding the genetic environment of *mcr-1.1*, the *mcr-1*-*pap2* (a gene encoding a putative PAP family transmembrane protein) element was detected in the two plasmids (Figure 3). However, the composite transposon (IS*Apl1*-IS*5D*-*pap2*-*mcr-1*-IS*Apl1*) (Figure 3) was only in pAMS-51-MCR1 suggesting the role of IS*Apl1* and its potential for horizontal gene transfer (Partridge et al., 2018). IS*Apl1* belongs to IS*30* family and encodes a DDE-type transposase (Partridge et al., 2018). It was first identified in the pig pathogen *Actinobacillus pleuropneumoniae* (Tegetmeyer et al., 2008) and was implicated in the acquisition and mobilization of *mcr-1* (Liu et al., 2016). The *mcr-1* genetic context, *pap2-mcr-1-ISApl1*, was observed in i) *E. coli* strain 803DBmcr plasmid 803-DB-mcr, isolated from human sample in China in 2017 (MH128771.1) (Figure 3), ii) *E. coli* strain ECZP248 plasmid pTBMCR401 isolated from chicken in China in 2017 (CP034785.1) (Figure 3; Chang et al., 2020), and iii) *E. coli* strain NDM132 plasmid pls1 recovered in China, (KX458104.1) (Figure 3). The plasmids pAMS-51-MCR1 and pAMS-73-MCR1 were effectively transferred by mating out assay to the recipient *E. coli* J53 strain with an efficiency of ∼2.7 × 10^–5^ and 1 × 10^0^ CFU/ml, respectively. PCR confirmed that transconjugants harbored *mcr-1.* The transconjugants carrying both the plasmids showed resistance to colistin (MICs = 2 or 4 μg/ml), and polymyxin B (MICs = 4 μg/ml) (Table 2). Additionally, the transconjugant *Ec*CAI51-Tc1 was resistant to CHL, and TET. It might be due to the transfer of both pAMS-51-MCR1 (which carry *cmlA1* conferring resistance to CHL) and pAMS-51-VR (which carry *tet*(A) conferring resistance to TET). In contrast, the other transconjugant *Ec*CAI73-TC3 was sensitive to CHL, and TET. However, the two transconjugant were slightly resistant to AMP which is suggested by the carriage of unknown β-lactamase on the transferred plasmids. To our knowledge, this is the first report of *mcr-1*-carrying IncP and IncX4 plasmids from human clinical *E. coli* isolates in Egypt.

### Analysis of the virulome of the two *mcr-1*-producing *Escherichia coli* isolates: Identification of two multireplicon virulence plasmids

Numerous virulence factors (VFs) have been detected chromosomally or on different plasmids contained within the two isolates, explaining its pathogenicity and virulence (Table 3). The chromosome of *E. coli Ec*CAI51 carried the following VFs: *air* (enteroaggregative immunoglobulin repeat protein), *chuA* (outer membrane hemin receptor), *eilA* (*Salmonella* HilA homolog), *gad* (glutamate decarboxylase), *ireA* (siderophore receptor), *papA_F20* (major pilin subunit F20), *papC* (outer membrane usher P fimbriae), and *terC* (tellurium ion resistance protein) while the chromosome of *E. coli Ec*CAI73 carried *gad*, *iha* (adherence protein), *mchB* (microcin H47 part of colicin H), *mchC* (MchC protein), *mchF* (ABC transporter protein MchF), and *terC*. Several MCR-1-producing *E. coli* isolates with virulence characters have been previously reported from Nepali patient admitted to an intensive-care unit in Qatar, and from retail poultry meat in Czech Republic (Forde et al., 2018; Kubelová et al., 2021).

**FIGURE 4:**
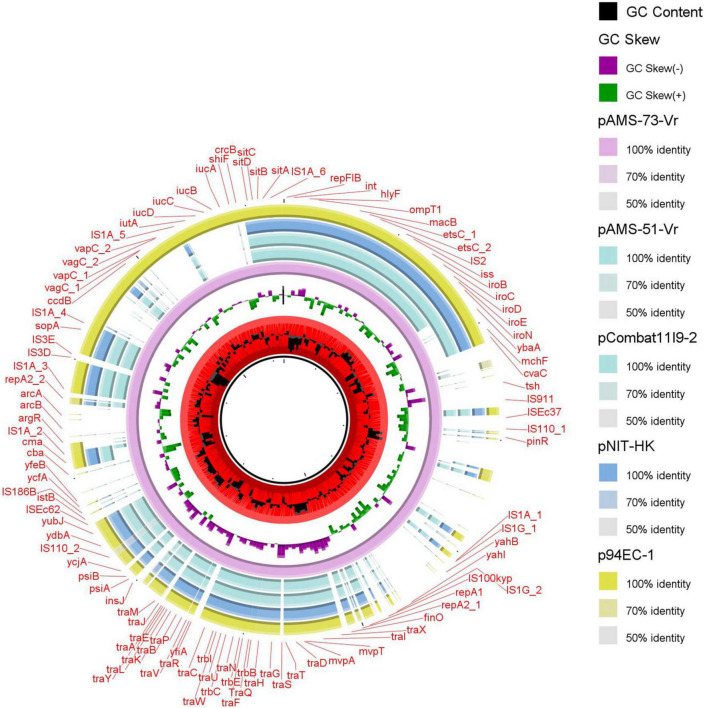
Schematic representation of the multireplicon virulence plasmids identified from the genome sequences of *E. coli* strains analyzed in this study. Four plasmid, pCombat11I9-2, pNIT-HK, and p94EC-1 (accession no. CP021728.1, MF474175.1, and CP047577.1, respectively) have been detected from NCBI GenBank and was included in the figure. The whole sequence of pAMS-73-Vr was used as the reference. The external ring represents the annotation of pAMS-73-Vr. The plasmids were included in the following order: pAMS-73-Vr (identified in this study), pAMS-51-Vr (identified in this study), pCombat11I9-2, pNIT-HK, and p94EC-1.

Two different multireplicon virulence plasmids (∼117 kb IncFII: IncFIB pAMS-51-Vr and ∼226 kb IncFIA: IncFIB: IncFIC: IncFII(K) pAMS-73-Vr) carrying several virulence genes were identified from the two isolates. Both the plasmids carried the *sitABCD* operon mediating resistance to hydrogen peroxide and catalyzing iron, manganese transport (Sabri et al., 2006), the Salmochelin siderophore *iroBCDE* operon mediating iron uptake and the *iroN* which act as siderophore receptor, mediating the utilization of the siderophore enterobactin (Russo et al., 2002). Additionally, pAMS-73-Vr carried the *iucABCD* operon and *iutA* mediating iron and aerobactin uptake (Torres et al., 2001). pAMS-73-Vr also carried the vacuolating autotransporter toxin (*vat* gene), which encourages the development of intracellular vacuoles causing cytotoxic effects related to those triggered by the *Helicobacter pylori* VacA toxin (Parreira and Gyles, 2003). The operons *sitABCD*, and *iucABCD* were previously described from *tet*(X7)-*mcr-1*/IncHI2 plasmids detected in *E. coli* isolates from poultry in Egypt and from plasmid pZM3 detected from an Algerian *Salmonella enterica* isolate (Harmer and Hall, 2020; Soliman et al., 2021). Numerous other virulence genes were detected from both isolates’ plasmids and were included into Table 3. A BLASTn search using the whole pAMS-51-Vr sequence query detected that it has > 99.9% identity with > 92% coverage to other virulence multireplicon plasmids detected from *E. coli* isolates as follow: (i) plasmid pCombat11I9-2 from strain Combat11I9 detected from urine in China (CP021728.1), (ii) plasmid pNIT-HK from strain J53/pNIT-HK isolated in Hong Kong (MF474175.1), and iii) plasmid p94EC-1 from strain 94EC isolated from human fecal sample in Singapore (CP047577.1) (Figure 4).

### Evolutionary relatedness of ST1011, and ST744 *mcr-1*-producing Egyptian clinical *Escherichia coli* isolates identified in this study

**FIGURE 5:**
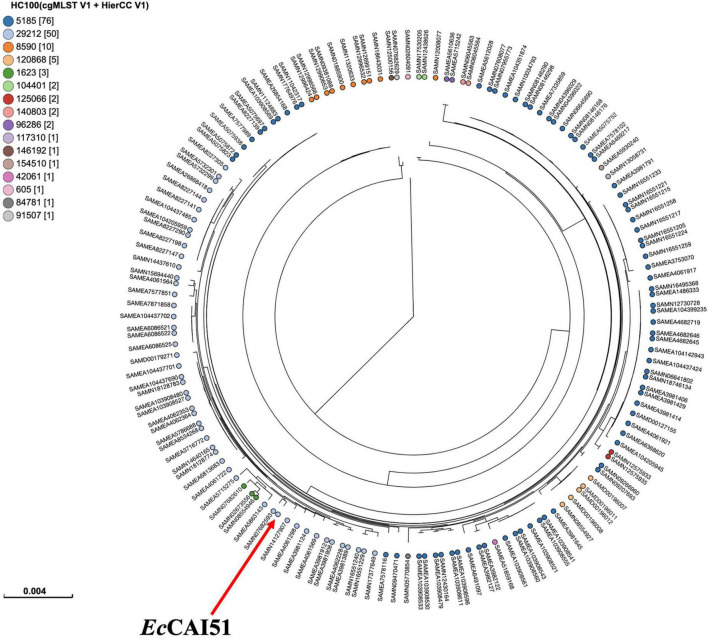
Phylogenetic analysis of ST1011 *Escherichia coli* isolate and other publicly available ST1011 *E. coli* isolates (*n* = 157) in Enterobase using single nucleotide polymorphisms (SNPs) and hierarchical clustering (HierCC) of core genome (cg) MLST.

**FIGURE 6:**
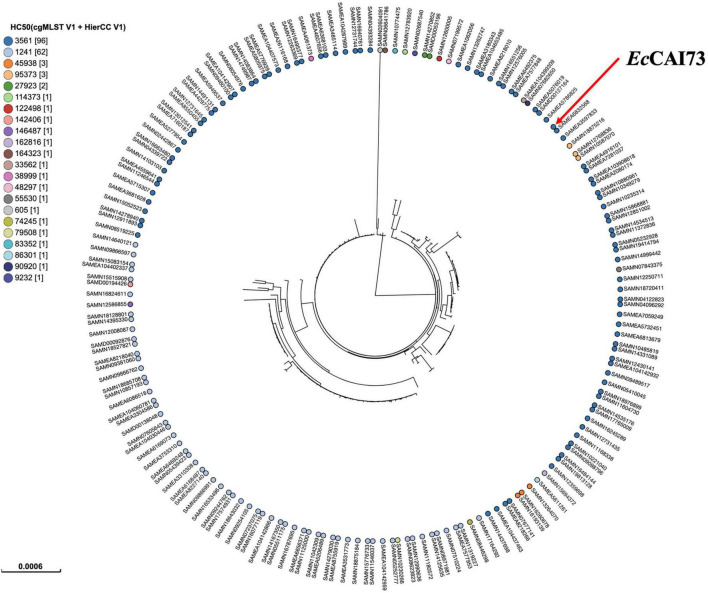
Phylogenetic analysis of ST744 *Escherichia coli* isolate *Ec*CAI73 and other publicly available ST744 *E. coli* isolates (*n* = 181) in Enterobase using single nucleotide polymorphisms (SNPs) and hierarchical clustering (HierCC) of core genome (cg) MLST.

Phylogenetic analysis was performed by comparing the genomes of our isolates *Ec*CAI51 (ST1011) and *Ec*CAI73 (ST744) to the publicly available *E. coli* genomes in Enterobase using SNPs and HierCC of cgMLST (Figures 5, 6). Based on differences of core genome loci among bacteria, different sets of hierarchical clusters (HCs) in Enterobase were designated to cluster bacterial genomes at higher resolution levels compared to the conventional MLST. At HC100 (≤ 100 allelic differences), the HC100 pattern (HC100| 29212) has been determined for our ST1011 isolate (*Ec*CAI51) and other ST1011 isolates from various countries including China, Japan, Vietnam, Bangladesh, Lebanon, and other European countries (Figure 5 and Supplementary Table 1). Likewise, our ST744 isolate (*Ec*CAI73) was clustered, and shared the same HC50| 3561 with no more than 50 allelic differences with other ST744 isolates from Thailand, the United States, Australia, Vietnam, Switzerland, Netherlands, Spain, and Portugal (Figure 6 and Supplementary Table 2). In our recently published reports, we have determined the clustering of *mcr*-producing *E. coli* from Poultry in Egypt with global *E. coli* lineages (Ramadan et al., 2021; Soliman et al., 2021), indicating the wide spread of antimicrobial-resistant clones and the requirement of implementing WGS-based phylogeny for disease surveillance and control interventions.

A recent study illustrated that the *mcr-1*/IncX4 plasmid (pHNSHP23) was stably maintained without an effect on the growth of their hosts conferring a fitness advantage and indicating the ability for an additional dissemination with or without the selection pressure of antibiotics (Wu et al., 2018). Furthermore, the *mcr-1*/IncP plasmids (pHNGDF1-1 and pHNGDF36-1) were quite stable and increased the biological fitness in the host (Lv et al., 2018). The IncP plasmids has a broad host range and a high conjugation efficiency which may accelerate the spreading of *mcr-1* across different hosts (Lv et al., 2018). The future perspective following this study might be as follow: I) assaying the stability of the two plasmids identified in this study, pAMS-51-MCR1 and pAMS-73-MCR, and II) analyzing the fitness costs of these two *mcr-1*-positive plasmids.

## Conclusion

To the best of our knowledge, this study presented the first complete genomic sequence of *mcr-1*-carrying IncP and IncX4 plasmids from human clinical *E. coli* isolates in Egypt. In addition, the study illustrated the *mcr-1* broad dissemination in diverse plasmids and dissimilar *E. coli* clones. A multireplicon virulence plasmid, named pAMS-73-Vr, carrying the operons *sitABCD*, *iroBCDE* and *iucABCD*/*iutA* was identified. Both the strains showed MDR phenotypes, which can be easily converted to extensive (XDR) or pan (PDR) drug-resistant phenotypes by horizontal gene transfer of any carbapenemase gene, particularly *bla*_NDM_ (highly prevalent in Egypt). Therefore, medical authorities must implement strict infection control policies and antimicrobial surveillance plans (including animals) to control the spread of such strains.

## Data availability statement

The datasets presented in this study can be found in online repositories. The names of the repository/repositories and accession number(s) can be found in the article/Supplementary material.

## Author contributions

AS and TaS designed and directed the study. AS performed identification of bacteria, screening and identifying of resistance genes, conjugation, plasmid analysis and typing, analyzed the WGS data, and drafted the manuscript. HR contributed to the genome analysis, data curation, and participated to the writing of the manuscript. SE carried out the collection of samples and bacterial isolation. HN, CJ, and RE-D conceived of the study, made the data analysis, and revised the manuscript. ToS made the data analysis. TaS participated in the discussion on the study design and finalized the manuscript. LY and JH participated in genomic DNA extraction, short-read library preparation, and sequencing. LY carried out long-read library preparation and sequencing, performed hybrid assembly of MiniSeq short reads and Nanopore long reads, and contributed to the genome analysis and performed DDBJ nucleotide sequence submission. MS and LY participated in the discussion on the study and revised the manuscript. All authors read and approved the final manuscript.

## Footnotes

1
http://www.eucast.org

2
https://enterobase.warwick.ac.uk/species/index/ecoli

3
https://dfast.nig.ac.jp/

4
http://www.genomicepidemiology.org/

5
http://brig.sourceforge.net/

6
http://mjsull.github.io/Easyfig/

7
https://blast.ncbi.nlm.nih.gov/Blast.cgi

8
https://isfinder.biotoul.fr/

9
https://enterobase.warwick.ac.uk/

## References

[B1] Borowiak M. Fischer J. Hammerl J. A. Hendriksen R. S. Szabo I. Malorny B. (2017). Identification of a novel transposon-associated phosphoethanolamine transferase gene, mcr-5, conferring colistin resistance in d-tartrate fermenting *Salmonella enterica* subsp. enterica serovar Paratyphi B. *J. Antimicrob. Chemother.* 72 3317–3324. 10.1093/jac/dkx327 28962028

[B2] Carattoli A. Bertini A. Villa L. Falbo V. Hopkins K. L. Threlfall E. J. (2005). Identification of plasmids by PCR-based replicon typing. *J. Microbiol. Methods* 63 219–228. 10.1016/j.mimet.2005.03.018 15935499

[B3] Carattoli A. Villa L. Feudi C. Curcio L. Orsini S. Luppi A. (2017). Novel plasmid-mediated colistin resistance mcr-4 gene in *Salmonella* and *Escherichia coli*, Italy 2013, Spain and Belgium, 2015 to 2016. *Euro Surveill.* 22:30589. 10.2807/1560-7917.ES.2017.22.31.30589 28797329 PMC5553062

[B4] Chang J. Tang B. Chen Y. Xia X. Qian M. Yang H. (2020). Two IncHI2 plasmid-mediated colistin-resistant *Escherichia coli* strains from the broiler chicken supply chain in Zhejiang Province, China. *J. Food Prot.* 83 1402–1410. 10.4315/JFP-20-041 32294180

[B5] Clermont O. Bonacorsi S. Bingen E. (2000). Rapid and simple determination of the *Escherichia coli* phylogenetic group. *Appl. Environ. Microbiol.* 66 4555–4558. 10.1128/AEM.66.10.4555-4558.2000 11010916 PMC92342

[B6] Clinical and Laboratory Standards Institute [CLSI] (2020). *Performance Standards for Antimicrobial Susceptibility Testing*, 30th Edn. Wayne, PA: CLSI.

[B7] Elbediwi M. Li Y. Paudyal N. Pan H. Li X. Xie S. (2019). Global burden of colistin-resistant bacteria: Mobilized colistin resistance genes study (1980–2018). *Microorganisms* 7:461. 10.3390/microorganisms7100461 31623244 PMC6843232

[B8] Elnahriry S. S. Khalifa H. O. Soliman A. M. Ahmed A. M. Hussein A. M. Shimamoto T. (2016). Emergence of plasmid-mediated colistin resistance gene mcr-1 in a clinical *Escherichia coli* isolate from Egypt. *Antimicrob. Agents Chemother.* 60 3249–3250. 10.1128/AAC.00269-16 26953204 PMC4862507

[B9] Forde B. M. Zowawi H. M. Harris P. N. A. Roberts L. Ibrahim E. Shaikh N. (2018). Discovery of mcr-1-mediated colistin resistance in a highly virulent *Escherichia coli* lineage. *mSphere* 3:e00486–18. 10.1128/mSphere.00486-18 30305321 PMC6180223

[B10] Harmer C. J. Hall R. M. (2020). The complete nucleotide sequence of pZM3, a 1970 FIA:FIB:FII plasmid carrying antibiotic resistance and virulence determinants. *Microb. Drug Resist.* 26 438–446. 10.1089/mdr.2019.0248 31718432

[B11] Hasman H. Hammerum A. M. Hansen F. Hendriksen R. S. Olesen B. Agersø Y. (2015). Detection of mcr-1 encoding plasmid-mediated colistin-resistant *Escherichia coli* isolates from human bloodstream infection and imported chicken meat, Denmark 2015. *Euro Surveill.* 20:30085. 10.2807/1560-7917.ES.2015.20.49.30085 26676364

[B12] Jousset A. B. Bernabeu S. Bonnin R. A. Creton E. Cotellon G. Sauvadet A. (2019). Development and validation of a multiplex polymerase chain reaction assay for detection of the five families of plasmid-encoded colistin resistance. *Int. J. Antimicrob. Agents* 53 302–309. 10.1016/j.ijantimicag.2018.10.022 30395987

[B13] Kubelová M. Koláčková I. Gelbíčová T. Florianová M. Kalová A. Karpíšková R. (2021). Virulence properties of mcr-1-positive *Escherichia coli* isolated from retail poultry meat. *Microorganisms* 9:308. 10.3390/microorganisms9020308 33540889 PMC7913130

[B14] Liassine N. Assouvie L. Descombes M. C. Tendon V. D. Kieffer N. Poirel L. (2016). Very low prevalence of MCR-1/MCR-2 plasmid-mediated colistin resistance in urinary tract Enterobacteriaceae in Switzerland. *Int. J. Infect. Dis.* 51, 4–5. 10.1016/j.ijid.2016.08.008 27544715

[B15] Liu Y. Y. Wang Y. Walsh T. R. Yi L. X. Zhang R. Spencer J. (2016). Emergence of plasmid-mediated colistin resistance mechanism MCR-1 in animals and human beings in China: A microbiological and molecular biological study. *Lancet Infect. Dis.* 16 161–168. 10.1016/S1473-3099(15)00424-7 26603172

[B16] Lu H. Wang C. Dong G. Xu C. Zhang X. Liu H. (2018). Prevalence and molecular characterization of *Escherichia coli* clinical isolates carrying mcr-1 in a Chinese teaching hospital from 2002 to 2016. *Antimicrob. Agents Chemother.* 62:e02623–17. 10.1128/AAC.02623-17 29987151 PMC6125521

[B17] Lu X. Zeng M. Xu J. Zhou H. Gu B. Li Z. (2019). Epidemiologic and genomic insights on mcr-1-harbouring *Salmonella* from diarrhoeal outpatients in Shanghai, China, 2006-2016. *EBioMedicine* 42 133–144. 10.1016/j.ebiom.2019.03.006 30905850 PMC6491383

[B18] Luo J. Li M. Zhou M. Hu Y. (2015). Characterization of a novel strain phylogenetically related to Kocuria rhizophila and its chemical modification to improve performance of microbial fuel cells. *Biosens. Bioelectron.* 69 113–120. 10.1016/j.bios.2015.02.025 25721974

[B19] Lv L. Cao Y. Yu P. Huang R. Wang J. Wen Q. (2018). Detection of mcr-1 gene among *Escherichia coli* isolates from farmed fish and characterization of mcr-1-bearing IncP plasmids. *Antimicrob. Agents Chemother.* 62:e02378–17. 10.1128/AAC.02378-17 29311062 PMC5826114

[B20] Parreira V. R. Gyles C. L. (2003). A novel pathogenicity island integrated adjacent to the thrW tRNA gene of avian pathogenic *Escherichia coli* encodes a vacuolating autotransporter toxin. *Infect. Immun.* 71 5087–5096. 10.1128/IAI.71.9.5087-5096.2003 12933851 PMC187369

[B21] Partridge S. R. Kwong S. M. Firth N. Jensen S. O. (2018). Mobile genetic elements associated with antimicrobial resistance. *Clin. Microbiol. Rev.* 31:e00088–17. 10.1128/CMR.00088-17 30068738 PMC6148190

[B22] Paveenkittiporn W. Kamjumphol W. Ungcharoen R. Kerdsin A. (2021). Whole-genome sequencing of clinically isolated carbapenem-resistant *Enterobacter*ales harboring mcr genes in Thailand, 2016–2019. *Front. Microbiol.* 11:586368. 10.3389/fmicb.2020.586368 33505364 PMC7829498

[B23] Poirel L. Kieffer N. Brink A. Coetze J. Jayol A. Nordmann P. (2016). Genetic features of MCR-1-producing colistin-resistant *Escherichia coli* isolates in South Africa. *Antimicrob. Agents Chemother.* 60 4394–4397. 10.1128/AAC.00444-16 27161623 PMC4914673

[B24] Ramadan H. Soliman A. M. Hiott L. M. Elbediwi M. Woodley T. A. Chattaway M. A. (2021). Emergence of multidrug-resistant *Escherichia coli* producing CTX-M, MCR-1, and FosA in retail food from Egypt. *Front. Cell. Infect. Microbiol.* 11:681588. 10.3389/fcimb.2021.681588 34327151 PMC8315045

[B25] Russo T. A. McFadden C. D. Carlino-MacDonald U. B. Beanan J. M. Barnard T. J. Johnson J. R. (2002). IroN functions as a siderophore receptor and is a urovirulence factor in an extraintestinal pathogenic isolate of *Escherichia coli*. *Infect. Immun.* 70 7156–7160. 10.1128/IAI.70.12.7156-7160.2002 12438401 PMC133021

[B26] Sabri M. Léveillé S. Dozois C. M. (2006). A SitABCD homologue from an avian pathogenic *Escherichia coli* strain mediates transport of iron and manganese and resistance to hydrogen peroxide. *Microbiology* 152 745–758. 10.1099/mic.0.28682-0 16514154

[B27] Soliman A. M. Maruyama F. Zarad H. O. Ota A. Nariya H. Shimamoto T. (2020a). Emergence of a multidrug-resistant *Enterobacter* hormaechei clinical isolate from Egypt co-harboring mcr-9 and blaVIM-4. *Microorganisms* 8:595. 10.3390/microorganisms8040595 32325973 PMC7232449

[B28] Soliman A. M. Zarad H. O. Nariya H. Shimamoto T. Shimamoto T. (2020b). Genetic analysis of carbapenemase-producing Gram-negative bacteria isolated from a university teaching hospital in Egypt. *Infect. Genet. Evol.* 77:104065. 10.1016/j.meegid.2019.104065 31634643

[B29] Soliman A. M. Ramadan H. Zarad H. Sugawara Y. Yu L. Sugai M. (2021). Coproduction of Tet(X7) conferring high-level tigecycline resistance, fosfomycin FosA4, and colistin Mcr-1.1 in *Escherichia coli* strains from chickens in Egypt. *Antimicrob. Agents Chemother.* 65:e02084–20. 10.1128/AAC.02084-20 33820767 PMC8315911

[B30] Stoesser N. Mathers A. J. Moore C. E. Day N. P. Crook D. W. (2016). Colistin resistance gene mcr-1 and pHNSHP45 plasmid in human isolates of *Escherichia coli* and *Klebsiella pneumoniae*. *Lancet Infect. Dis.* 16 285–286. 10.1016/S1473-3099(16)00010-4 26774239

[B31] Tartor Y. H. El-Aziz A. Khairy N. Gharieb R. El Damaty H. M. Enany S. (2021a). Whole genome sequencing of Gram-negative bacteria isolated from bovine mastitis and raw milk: The first emergence of colistin mcr-10 and fosfomycin fosA5 resistance genes in *Klebsiella pneumoniae* in Middle East. *Front. Microbiol.* 12:770813. 10.3389/fmicb.2021.770813 34956131 PMC8692987

[B32] Tartor Y. H. Gharieb R. El-Aziz A. Norhan K. El Damaty H. M. Enany S. (2021b). Virulence determinants and plasmid-mediated colistin resistance mcr genes in Gram-negative bacteria isolated from bovine milk. *Front. Cell. Infect. Microbiol.* 11:761417. 10.3389/fcimb.2021.761417 34888259 PMC8650641

[B33] Tegetmeyer H. E. Jones S. C. Langford P. R. Baltes N. (2008). ISApl1, a novel insertion element of Actinobacillus pleuropneumoniae, prevents ApxIV-based serological detection of serotype 7 strain AP76. *Vet. Microbiol.* 128 342–353. 10.1016/j.vetmic.2007.10.025 18065168

[B34] Torres A. G. Redford P. Welch R. A. Payne S. M. (2001). TonB-dependent systems of uropathogenic *Escherichia coli*: Aerobactin and heme transportand TonB are required for virulence in the mouse. *Infect. Immun.* 69 6179–6185. 10.1128/IAI.69.10.6179-6185.2001 11553558 PMC98749

[B35] Wick R. R. Judd L. M. Gorrie C. L. Holt K. E. (2017). Unicycler: Resolving bacterial genome assemblies from short and long sequencing reads. *PLoS Comput. Biol.* 13:e1005595. 10.1371/journal.pcbi.1005595 28594827 PMC5481147

[B36] Wu R. Yi L. X. Yu L. F. Wang J. Liu Y. Chen X. (2018). Fitness advantage of mcr-1-bearing IncI2 and IncX4 plasmids in vitro. *Front. Microbiol.* 9:331. 10.3389/fmicb.2018.00331 29535696 PMC5835064

[B37] Xavier B. B. Lammens C. Ruhal R. Kumar-Singh S. Butaye P. Goossens H. (2016). Identification of a novel plasmid-mediated colistin-resistance gene, mcr-2, in *Escherichia coli*, Belgium, June 2016. *Euro Surveill.* 21:27. 10.2807/1560-7917.ES.2016.21.27.30280 27416987

[B38] Yin W. Li H. Shen Y. Liu Z. Wang S. Shen Z. (2017). Novel plasmid-mediated colistin resistance gene mcr-3 in *Escherichia coli*. *mBio* 8:e00543–17. 10.1128/mBio.00543-17 28655818 PMC5487729

[B39] Zakaria A. S. Edward E. A. Mohamed N. M. (2021). Genomic insights into a colistin-resistant uropathogenic *Escherichia coli* strain of O23:H4-ST641 lineage harboring mcr-1.1 on a conjugative IncHI2 plasmid from Egypt. *Microorganisms* 9:799. 10.3390/microorganisms9040799 33920265 PMC8069611

[B40] Zhou Z. Alikhan N. F. Mohamed K. Fan Y. Achtman M. Agama Study Group (2020). The EnteroBase user’s guide, with case studies on *Salmonella* transmissions, Yersinia pestis phylogeny, and *Escherichia* core genomic diversity. *Genome Res.* 30 138–152. 10.1101/gr.251678.119 31809257 PMC6961584

